# Can balloon-assisted enteroscopy predict disease outcomes in patients with small-bowel Crohn’s disease?

**DOI:** 10.1186/s12876-023-02892-3

**Published:** 2023-09-27

**Authors:** Ji Eun Na, Sung Noh Hong, Ji Eun Kim, Eun Ran Kim, Young-Ho Kim, Dong Kyung Chang

**Affiliations:** 1 Division of Gastroenterology, Department of Medicine, Samsung Medical Center, Sungkyunkwan University School of Medicine, 81 Irwon-ro, Gangnam-gu, Seoul, 06351 Republic of Korea; 2 Department of Medicine, Inje University Haeundae Paik Hospital, Busan, Korea

**Keywords:** Small bowel Crohn’s disease, Balloon-assisted enteroscopy, Simple endoscopic score for Crohn’s disease, Predictive value for the risk of surgery

## Abstract

There are limited studies on the endoscopic assessment of disease activity using balloon-assisted enteroscopy (BAE) and its predictive role for long-term outcomes of patients with small bowel Crohn’s disease (CD). We sought to investigate the value of BAE as a predictor of long-term outcomes in patients with small-bowel CD. A total of 111 patients with small-bowel CD whose endoscopic disease activity was assessed using BAE based on the small-bowel simple endoscopic score for Crohn’s disease (small-bowel SES-CD) at Samsung Medical Center were retrospectively selected from January 2014 to August 2020. The outcome was an evaluation of the risk of surgery according to a small-bowel SES-CD of 0–6 vs. ≥ 7 and endoscopic findings (presence of any ulcer and degree of stricture) using the Cox proportional hazards model. The risk of surgery was significantly increased in patients with a small-bowel SES-CD of ≥ 7 compared to a small-bowel SES-CD of 0–6 [hazard ratio (HR) 6.31; 95% confidence interval (CI) 1.48–26.91; *p* = 0.013]. In addition, the risk of surgery was significantly increased in patients with stenosis with “cannot be passed” compared to the cases without stenosis (HR 12.34; 95% CI 1.66–91.92; *p* = 0.014), whereas there was no significance in any ulcer. The present study demonstrated the role of BAE in the endoscopic assessment of disease activity and its predictive value for the risk of surgery in small-bowel CD patients. Further optimization of BAE utilization for the assessment of disease activity is warranted in clinical practice.

## Introduction

Endoscopic healing (EH) is a treatment target in Crohn’s disease (CD) and is associated with favorable outcomes such as long-term clinical remission and decreased risk of undergoing surgery [1, 2]. Hence, the endoscopic assessment of disease activity is a crucial aspect of clinical practice using assessment indices such as the Crohn’s disease endoscopic index of severity (CDEIS) or the simple endoscopic score in Crohn’s Disease (SES-CD) that integrates the degree of ulceration and bowel damage (stenosis) in CD patients [3–5].

Previous studies that conducted an endoscopic assessment of disease activity by ileocolonoscopy in CD patients were limited in application to patients with small-bowel CD due to the inaccessibility of the small bowel beyond the terminal ileum (TI). CD involves up to 80% of the small bowel, including the ileocolon [6, 7], whereas small bowel lesions skipping the terminal ileum can be missed by ileocolonoscopy [8]. With the advent of balloon-assisted enteroscopy (BAE), the evaluation of and intervention for small-bowel lesions are now more readily possible [9–11]. Hence, it is necessary to validate whether the endoscopic assessment of disease activity by BAE utilizing the current scoring systems (SES-CD or CDEIS) is applicable to patients with small-bowel CD.

In addition, since mucosal healing of the small bowel is more difficult than that of the colon after current treatment [12], there is a need to identify the association between endoscopic findings and the long-term outcomes in patients with small-bowel CD. The identification of endoscopic findings that can predict long-term outcomes allows physicians to pay attention to patients who need therapeutic interventions. Previously, patients with severe endoscopic colonic lesions (coalescent ulcerations covering more than 10% of the mucosal area of at least one segment of the colon) based on ileocolonoscopy had increased surgical risks [13], whereas, in the biologics era, stenosis rather than severe inflammation has emerged as a predictive factor for the risk of surgery [14]. Until now, reports on the endoscopic assessment of disease activity based on BAE and predictions for the risk of surgery in small-bowel CD patients have been limited [15]. Therefore, the objective of this study was to estimate the risk of surgery in small-bowel CD patients based on the BAE findings using a small-bowel SES-CD, a modified adaptation of the original SES-CD.

## Materials and methods

### Patients

**Fig. 1:**
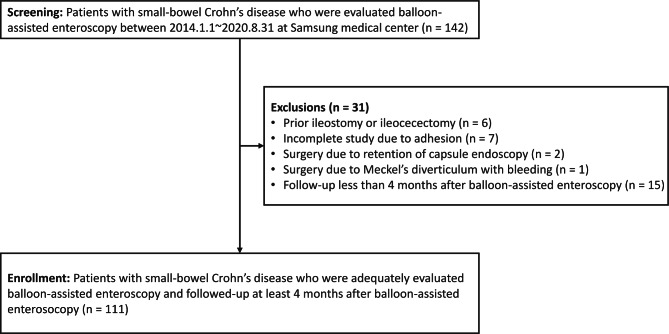
Diagram of patient selection

The patients diagnosed with small-bowel CD and evaluated by BAE for initial or consecutive assessments of disease activity were retrospectively screened at Samsung Medical Center, Seoul, Korea, from January 2014 to August 2020 (n = 142). CD was diagnosed based on the practice guidelines [16]. We excluded 31 patients based on the following exclusion criteria: (1) prior ileostomy or ileocecectomy (n = 6), (2) incomplete study due to paradoxical reaction to sedatives, adhesion, bleeding, or any other reasons (n = 7), (3) surgery due to the retention of the capsule endoscopy (n = 2), (4) surgery due to Meckel’s diverticulum with bleeding (n = 1), or (5) last hospital follow-up less than 4 months after BAE (n = 15). Finally, 111 patients with small-bowel CD who were adequately evaluated by BAE and followed more than 4 months after BAE were selected (Fig. 1). This study was approved by the Institutional Review Board of Samsung Medical Center (IRB File Number: 2020-08-146).

### Balloon-assisted enteroscopy, assessment index, and follow-up

The SIF-Q260 (Olympus, Tokyo, Japan) and single-use hydrophilic-coated silicone splinting tube (ST-SB1, Olympus, Tokyo, Japan) were used for BAE. The examination was performed under conscious sedation. All BAEs were performed by two physicians who had sufficient clinical experience with BAE and CD patients. The procedure was performed via the mouth (anterograde) and/or via the anus (retrograde), and the push-and-pull technique was adopted. The enteroscope was advanced through the small bowel by alternately inflating and deflating the balloon, and the small bowel was moved toward the endoscopist by pleating the intestine over a sliding tube in the same manner a curtain is pulled over a rod.

Since the implementation of BAE at our institution, we have developed a standardized protocol and report form to ensure consistency in reporting BAE procedure. We refer to the patient’s previous cross-sectional image to get a rough idea of the location and extent of the small bowel lesions and choose between a transoral or transanal approach. Distinguishing the segments of the small bowel anatomically and performing total enteroscopy using BAE is challenging since it is not a fixed organ like the colon and its long length. So, we classify the jejunum into proximal to mid (up to 80 cm from the Treitz ligament) and distal jejunum (beyond 80 cm from the Treitz ligament), taking into consideration the reachable location with BAE. For the ileum, we divide it into segments based on the observable areas using BAE, namely proximal (> 100 cm from the ileocecal valve), mid (50–100 cm from the ileocecal valve), distal (10–50 cm from the ileocecal valve), and terminal ileum (10 cm from the ileocecal valve). This differentiation of segments helps in better understanding among the medical staff during the interpretation of endoscopic reports.

Our institution has applied a format of endoscopic reports based on SES-CD in small-bowel CD patients. SES-CD for each segment of the small bowel provides a systematic evaluation of disease activity based on BAE findings. In this study, we denominate with small-bowel SES-CD as an objective index for the endoscopic assessment of disease activity. Two physicians retrospectively reviewed the scored small-bowel SES-CD based on BAE images and endoscopic reports. The small-bowel SES-CD per patient was adopted based on one of the most severe segments among proximal to mid/distal jejunum or proximal/mid/distal/terminal ileum. In other words, we reviewed the endoscopic disease activity for all achievable segments and chose the most severe segment in the per-patient analysis. The small-bowel SES-CD was calculated by four endoscopic variables that were the same as those in the original SES-CD and scored from 0 to 3 based on the presence and size of ulcers (none = score 0; diameter 0.1–0.5 cm = score 1; 0.5–2 cm = score 2; >2 cm = score 3); the extent of the ulcerated surface (none = 0; <10%=1; 10–30%=2; >30%=3); the extent of the affected surface (none = 0; <50%=1; 50–75%=2; >75%=3); and the presence and type of narrowing (none = 0; single, can be passed = 1; multiple, can be passed = 2; cannot be passed = 3). The small-bowel SES-CD values ranged from 0 to 12 and were divided into of 0–6 vs. ≥ 7. Endoscopic disease activity was also evaluated according to the presence of any ulcer and stenosis as well as the small-bowel SES-CD. Any ulcer with “yes” was defined as a definitive ulcer of 0.5 cm or more, excluding aphthous ulcers. Stenosis was classified according to severity as “none”, “single or multiple, can be passed”, or “cannot be passed” using the original SES-CD categories.

Endoscopic balloon dilatation (EBD) was performed when insertion was not possible because of stenosis. Before the EBD, the stricture characteristics were evaluated based on endoscopic findings and by using fluoroscopy after the infusion of radiocontrast dye. When stenosis occurred in two or more locations, the EBD was intended for all the evaluated stenoses. But, if the stenosis was accompanied by deep ulceration and was judged to have a risk of complications such as perforation, EBD was not performed. If further insertion is limited due to stenosis, the evaluation is carried out for each observable segment.

The index date was defined as the date of BAE. After BAE, the patients were usually monitored at 1–3 month intervals. The clinical response, which was the Harvey Bradshaw Index (HBI) and laboratory data that included hemoglobin, albumin, erythrocyte sedimentation rate (ESR), and C-reactive protein (CRP) levels, were checked at each visit. Additional cross-sectional imaging or BAE was performed as needed during the follow-up. The end of follow-up was defined as the date of surgery or the last hospital visit, whichever came first (reference date: December 31, 2020). Abdominal surgery was defined as the performance of small bowel resection and anastomosis or ileocecectomy during the follow-up period.

### Outcomes and covariates

The endoscopic assessment of disease activity was as follows: (i) small-bowel SES-CD: 0–6 vs. ≥ 7, (ii) any ulcer: no vs. yes, and (iii) stenosis: no vs. single or multiple, can be passed vs. cannot be passed. The primary outcome was the evaluation of the risk of surgery according to the small-bowel SES-CD 0–6 vs. ≥ 7. The secondary outcome was the assessment of the association of the BAE finding (presence of any ulcer and degree of stricture) with surgery. The tertiary outcome was to identify the predictors of surgery in the overall patient cohort.

The following covariates were collected by retrospective review of the medical record based on the index date (the date of BAE): gender, age at diagnosis, age at BAE, disease duration, location of disease, the behavior of the disease, perianal disease, history of smoking, history of surgery [only including small bowel resection and anastomosis (SBRS) and segmental colectomy], HBI, laboratory data (hemoglobin, albumin, ESR, and CRP), concomitant treatment (immunomodulators or biologics), and balloon dilatation at BAE. The biologics included anti-tumor necrosis factor (TNF) agents (infliximab and adalimumab), vedolizumab, and ustekinumab.

### Statistical analysis

For the comparison of the baseline characteristics between the no surgery group and the surgery group, the continuous variables were analyzed using the Wilcoxon rank-sum test, and the categorical variables were analyzed using the chi-squared test or Fisher’s exact test. The risk of surgery was analyzed using the Cox proportional hazards model, and the cumulative surgery-free survival rate was analyzed using the Kaplan Meier curve with a log-rank test according to the small-bowel SES CD of 0–6 vs. ≥ 7, or presence or absence of ulcers, or stenosis. To find an optimal cut-off point for predicting surgical risk using the small-bowel SES-CD, we analyzed the receiver operating characteristic (ROC) curve and calculated the corresponding Youden index. A cut-off value 7 was selected, as it yielded the highest Youden index. A subgroup analysis of surgical outcomes was performed in patients classified as biologics-naïve or concomitant to biologics in the overall cohort. To identify the predictors of surgery, univariate and multivariate Cox regression analysis was conducted. Candidate predictors with *p*-values of ˂ 0.05 in the univariate analysis were included in the multivariate analysis. A *p*-value of < 0.05 was considered statistically significant. Statistical analysis was performed using IBM SPSS statistics 27.0.

## Results

### Patients and baseline characteristics of the study population

Of the 111 small-bowel CD patients included in the analysis, 23 patients underwent surgery during the follow-up. The baseline characteristics are shown in Table 1 and compared between the non-surgery and surgery groups. Non-surgery and surgery groups were comparable regarding gender; age at diagnosis; location of disease; history of smoking; the proportion of prior surgeries (SBRA and segmental colectomy); hemoglobin, albumin, and ESR levels; and concomitant treatment (immunomodulators or biologics). The surgery group had an older age at BAE, a longer duration of disease, a higher proportion of stricturing or penetrating behavior, a higher proportion of perianal disease, a higher proportion of moderate-to-severe HBI, higher CRP levels, and a higher proportion of balloon dilation at BAE than those in the non-surgery group.

**Table 1:** Baseline characteristics

Variable	Overall (*n =* 111)	Non-surgery (*n =* 88)	Surgery (*n =* 23)	*p*-value^a^
Gender				0.576
Male	87 (78.4)	70 (79.5)	17 (73.9)	
Female	24 (21.6)	18 (20.5)	6 (26.1)	
Age at diagnosis				0.682
A1: < 16 years	5 (4.5)	5 (5.7)	0 (0.0)	
A2: 16–40 years	81 (73.0)	64 (72.7)	17 (73.9)	
A3: >40 years	25 (22.5)	19 (21.6)	6 (26.1)	
Age at BAE, years^†^	33 (25, 42)	32 (24, 41)	35 (32, 49)	0.046
Disease duration, years^†^	1 (0, 6)	0 (0, 5)	5 (0, 10)	0.010
Location				0.834
L1: ileal	81 (73.0)	63 (71.6)	18 (78.3)	
L3: ileocolonic	29 (26.1)	24 (27.3)	5 (21.7)	
Upper disease	21 (18.9)	16 (18.2)	5 (21.7)	0.766
Behavior				0.002
B1: inflammatory	37 (33.3)	36 (40.9)	1 (4.3)	
B2: stricturing	56 (50.5)	41 (46.6)	15 (65.2)	
B3: penetrating	18 (16.2)	11 (12.5)	7 (30.4)	
Perianal disease	16 (14.1)	9 (10.2)	7 (30.4)	0.022
Current or Ex-smoking	27 (24.3)	19 (21.6)	8 (34.8)	0.189
SBRA or segmental colectomy	23 (20.7)	16(18.2)	7 (30.4)	0.248
Harvey-Bradshaw Index ^†^				0.041
Remission	53 (47.7)	45 (51.1)	8 (34.8)	
Mild	28 (25.2)	24 (27.3)	4 (17.4)	
Moderate to severe	30 (27.0)	19 (21.6)	11 (47.8)	
Laboratory data^†^				
Hemogloblin, g/dL	12.2 (10.3, 13.4)	12.4 (10.4, 13.4)	11.7 (8.9, 12.8)	0.166
Albumin, g/dL	4.2 (3.5, 4.6)	4.3 (3.6, 4.6)	3.9 (3.4, 4.3)	0.073
ESR, mg/L	10.0 (4.0, 21.0)	9.0 (4.0, 18.0)	14.0 (5.0, 34.0)	0.055
CRP, mg/L	0.2 (0.1, 0.6)	0.1 (0.1, 0.5)	0.4 (0.2, 1.2)	0.004
Concomitant treatment				
Immunomodulators	42 (37.8)	32 (36.4)	10 (43.5)	0.531
Biologics	31 (27.9)	21 (23.9)	10 (43.5)	0.062
Balloon dilatation at BAE	43 (38.7)	28 (31.8)	15 (65.2)	0.003
Follow-up duration, months^†*^	23 (15, 33)	23 (14, 32)	23 (15, 33)	0.456

BAE, balloon-assisted enteroscopy; CD, Crohn’s disease, CI, confidence interval; CRP, C-reactive protein; ESR, erythrocyte sedimentation rate; HR, hazard ratio; SBRA, small bowel resection and anastomosis

Values are expressed as *n* (%) unless otherwise specified

^†^Value is median (IQR, interquartile range)

*Between the BAE date and the last follow-up

^a^*p*-value calculated using Wilcoxon rank sum test for continuous variables and chi-square test or Fisher’s exact test for categorical variables for overall data

### Surgical outcomes

**Fig. 2:**
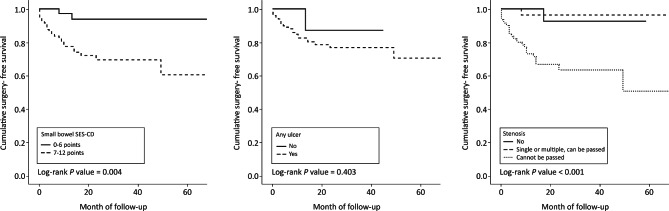
Cumulative surgery-free survival in the overall patients

The median (IQR) follow-up duration from the date of BAE to the last hospital visit was 23 months (15, 33). Among the overall 111 patients, 2 of 38 patients in the small-bowel SES-CD 0–6 group vs. 21 of 73 patients in the small-bowel SES-CD ≥ 7 group underwent surgery and the risk of surgery was significantly increased in the small-bowel SES-CD ≥ 7 group compared to the small-bowel SES-CD 0–6 group (HR 6.31; 95% CI: 1.48–26.91; *p* = 0.013). One of 10 patients with “no” for any ulcer vs. 22 of 101 patients with “yes” for any ulcer underwent surgery, and there was no significant association between any ulcer and the risk of surgery (HR 2.29; 95% CI 0.31–17.06; *p* = 0.419). Regarding stenosis, one of 28 patients with “no”, one of 21 patients with “single or multiple, can be passed,” and 21 of 62 patients with “cannot be passed” underwent surgery, indicating a significantly increased risk of surgery in patients with stenosis that “cannot be passed” compared to the cases without stenosis (HR 12.34; 95% CI 1.66–91.92, *p* = 0.014) (Table 2). The cumulative surgery-free survival rate was significantly lower in the small-bowel SES-CD ≥ 7 group compared to the small-bowel SES-CD 0–6 group (log-rank *p* = 0.004), and in those with stenosis that “cannot be passed” compared to categories of “no” and “single or multiple, can be passed” (log-rank *p* = 0.001), whereas no difference according to any ulcer was found (log-rank *p* = 0.403) (Fig. 2).

**Table 2:** Surgical outcomes according to the endoscopic assessment of disease activity

Overall		Total N	Surgery, *n* (%)	HR (95% CI)	*p*-value^a^
Small-bowel SES-CD	0–6	38	2 (5.3)	1	
	7–12	73	21 (28.8)	6.31 (1.48–26.91)	0.013
Any ulcer	No	10	1 (10.0)	1	
	Yes	101	22 (21.8)	2.29 (0.31–17.06)	0.419
Stenosis	No	28	1 (3.6)	1	
	Single or multiple, can be passed	21	1 (4.8)	1.41 (0.09–22.55)	0.808
	Cannot be passed	62	21 (33.9)	12.34 (1.66–91.92)	0.014
Biologics naïve					
Small-bowel SES-CD	0–6	27	1 (3.7)	1	
	7–12	53	12 (22.6)	6.94 (0.90-53.35)	0.063
Any ulcer	No	3	0 (0.0)	1	
	Yes	77	13 (16.9)	21.42 (0.00-)	0.621
Stenosis	No	21	1 (4.8)	1	
	Single or multiple, can be passed	18	1 (5.6)	1.18 (0.07–18.92)	0.905
	Cannot be passed	41	11 (26.8)	6.85 (0.88–53.16)	0.066
Concomitant biologics					
Small-bowel SES-CD	0–6	11	1 (9.1)	1	
	7–12	20	9 (45.0)	5.02 (0.62–40.36)	0.130
Any ulcer	No	7	1 (14.3)	1	
	Yes	24	9 (37.5)	2.49 (0.31–19.92)	0.391
Stenosis	No	7	0 (0.0)	1	
	Single or multiple, can be passed	3	0 (0.0)	1.04 (0.00-)	0.995
	Cannot be passed	21	10 (47.6)	43.90 (0.07-)	0.254

CI, confidence interval; HR, hazard ratio; SES-CD, simple endoscopic score for Crohn’s disease

^a^*p*-value calculated using Cox proportional hazard model

**Fig. 3:**
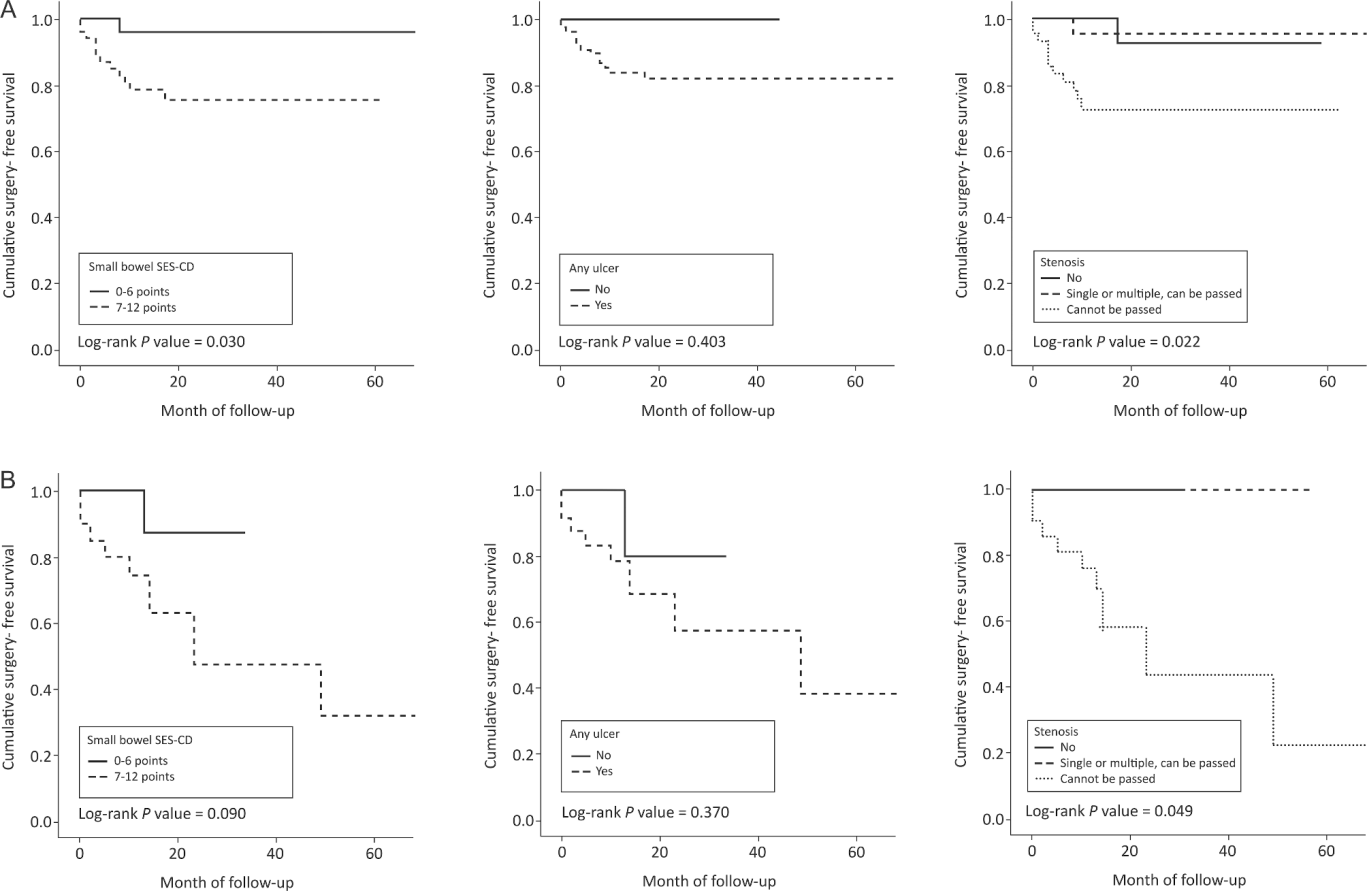
Cumulative surgery-free survival in biologics-naïve patients (**A**) and those with concomitant biologics use (**B**)

In the subgroup of biologics-naïve patients, those with a small-bowel SES-CD of 0–6 vs. ≥ 7 (HR 6.94; 95% CI: 0.90–53.35, *p* = 0.063), any ulcer (HR 21.42; 95% CI 0.00-, *p* = 0.621), and stenosis (HR 6.85; 95% CI 0.88–53.16; *p* = 0.066) did not show a statistical significance in the risk of surgery (Table 2). The cumulative surgery-free survival rate was significantly lower in the small-bowel SES-CD ≥ 7 group compared to the small-bowel SES-CD 0–6 group (log-rank *p* = 0.030) and in those with stenosis that “cannot be passed” compared to categories of “no” and “single or multiple, can be passed” (log-rank *p* = 0.022), whereas no difference according to any ulcer was found (log-rank *p* = 0.403) (Fig. 3A).

In the subgroup of patients with concomitant biologics treatment, a small-bowel SES-CD of 0–6 vs. ≥ 7 (HR 5.02; 95% CI 0.62–40.36; *p* = 0.130), any ulcer (HR 2.49; 95% CI 0.31–19.92; *p* = 0.391), and stenosis that “cannot be passed” (HR 43.90; 95% CI 0.07-; *p* = 0.254) did not show a statistical difference in the risk of surgery (Table 2). The cumulative surgery-free survival rate was significantly lower only in patients with stenosis that “cannot be passed” compared to categories of “no” and “single or multiple, can be passed” (log-rank *p* = 0.049) (Fig. 3B).

### Predictors of surgery

The candidate predictors with *p*-values of < 0.05 in univariate analysis were included in the multivariate analysis as follows: a small-bowel SES-CD of 0–6 vs. ≥ 7; duration of CD; perianal disease; CRP levels; and balloon dilatation at BAE. The multivariate analysis showed that a small-bowel SES-CD of 0–6 vs. ≥ 7 (HR 7.89; 95% CI 1.77–35.05; *p* = 0.007) and duration of CD (HR 1.17; 95% CI; 1.07–1.29; *p* = 0.001) was an independent predictor associated with an increased risk of surgery (Table 3).

**Table 3:** Predictors of the surgery in cox regression analysis in overall patients

Variables	Univariate		Multivariate	
	HR (95% CI)	*p*-value^a^	HR (95% CI)	*p*-value^a^
Small-bowel SES-CD				
0–6	1		1	
≥ 7	6.31 (1.48–26.91)	0.013	7.89 (1.77–35.05)	0.007
Gender				
Male	1			
Female	1.43 (0.56–3.65)	0.450		
Age at diagnosis	1.01 (0.99–1.04)	0.456		
Duration of CD (years)	1.13 (1.05–1.22)	0.002	1.17 (1.07–1.29)	0.001
Upper disease	1.06 (0.39–2.87)	0.904		
Perianal disease	3.89 (1.56–9.66)	0.003	1.75 (0.63–4.87)	0.286
Current or Ex-smoking	1.72 (0.73–4.10)	0.218		
SBRA and segmental colectomy	1.82 (0.75–4.43)	0.186		
Harvey-Bradshaw Index				
Remission	1	0.065		
Mild	0.99 (0.30–3.30)	0.987		
Moderate to severe	2.71 (1.09–6.75)	0.032		
Hemogloblin, g/dL	0.87 (0.72–1.07)	0.181		
Albumin, g/dL	0.61 (0.37–1.01)	0.053		
ESR, mg/L	1.01 (0.99–1.02)	0.257		
CRP, mg/L	1.59 (1.03–2.47)	0.038	1.75 (0.93–3.28)	0.081
Concomitant immunomodulators	1.43 (0.62–3.26)	0.402		
Concomitant biologics	2.11 (0.92–4.81)	0.077		
Balloon dilatation at BAE	3.27 (1.38–7.71)	0.007	2.27 (0.69–7.47)	0.180

BAE, balloon-assisted enteroscopy; CD, Crohn’s disease, CI, confidence interval; CRP, C-reactive protein; ESR, erythrocyte sedimentation rate; HR, hazard ratio

^a^*p*-value calculated using univariable and multivariable Cox regression analysis

## Discussion

In this study, a small-bowel SES-CD ≥ 7 and stenosis that “cannot be passed” were associated with an increased risk of surgery, and a small-bowel SES-CD of ≥ 7 was an independent predictor of surgery in patients with small-bowel CD. In the subgroup of biologics-naïve patients, the cumulative surgery-free survival rate was low in patients with a small-bowel SES-CD of ≥ 7 and stenosis that “cannot be passed.” In the subgroup of patients with concomitant biologics use, the cumulative surgery-free survival rate was low in those with stenosis that “cannot be passed”, whereas there was no significant difference between an SES-CD of 0–6 and ≥ 7. The small-bowel SES-CD of ≥ 7 and disease duration were identified as predictive factors for surgery. These findings suggest that the endoscopic assessment of disease activity using BAE was valuable in that the risk of surgery can be predicted based on the BAE findings.

This study was the first to demonstrate the value of BAE in patients with small-bowel CD using a small-bowel SES-CD that was modified for evaluating small bowel lesions. Previous studies on the association of endoscopic healing and long-term outcomes in CD patients have been limited to application in small-bowel CD because the endoscopic assessment of disease activity was conducted by ileocolonoscopy, not by BAE [5, 17]. Even in a study based on BAE, there were only fragmentary comparisons according to active ulcers or the presence of strictures [15]. The strength of our study was that we introduced a reasonable tool, small-bowel SES-CD, for the endoscopic assessment of disease activity. The usefulness of small-bowel SES-CD is that it is familiar and easy to apply since each variable in the original SES-CD was used as it is, and it is based on one of the most severely diseased segments in the jejunum or ileum. In addition, this study provided a comparison of surgical outcomes according to the presence or absence of ulcers and stenosis separately. And a subgroup analysis in biologics-naïve patients or those with concomitant biologics use was performed to determine if there was a difference in the risk of surgery in the biologics era.

In previously reported data based on BAE for small-bowel CD patients [12, 15, 18, 19], the SES-CD active score (SES-CDa; endoscopic assessment exclusively for inflammation, excluding stenosis) ≥ 5 was associated with an increased risk of surgery in small-bowel CD patients with clinical and laboratory remission status [15]. In terms of clinical usefulness, the above study might have limitations in that inflammation (ulcer) and bowel damage (stenosis) were not comprehensively evaluated, and stenosis, defined as a diameter of ≤ 15 mm, was not stratified by severity. Based on our findings, even if the SES-CDa is less than 5, it can be associated with an increased risk of surgery if the lesion is accompanied by stenosis that “cannot be passed”, consequently yielding a small-bowel SES-CD of ≥ 7. If patients with an increased risk of surgery can be predicted using BAE, it could be used to raise caution and highlight the need for an intervention and would be a desirable tool for supporting the management of patients with small-bowel CD.

Although stenosis was not included in the multivariate analysis considering multicollinearity, stenosis that “cannot be passed” was a factor associated with an increased risk of surgery in the overall cohort and showed significantly low cumulative surgery-free survival rates in the overall cohort and subgroups consistently. This observation was consistent with a previous report that stenosis assessed by ileocolonoscopy was an independent predictor of abdominal surgery in the biologics era [14]. To date, BAE has been considered the most reliable tool for the detection of stenosis and the evaluation of its severity compared to magnetic resonance enterography with reduced sensitivity [19], and has the advantage that interventions such as EBD can be performed simultaneously. These findings support the role of BAE in the assessment of the degree and treatment of bowel damage.

A limitation of this study was that cross-sectional imaging was only performed in some of the patients (85 of 111 patients) within 3 months based on the date of BAE, so the covariates related to cross-sectional images were not included. Our study was designed as a single-center study. Therefore, further validation of the role of BAE is warranted for the general application of our observations. The number of subgroup patients was small, so the statistical power may have been insufficient to demonstrate the association of the endoscopic assessment of disease activity with the risk of surgery in the subgroup analysis. For example, there were no surgical events in the subgroup of biologics-naïve patients with “no” for any ulcer, and there were no surgical events in the subgroup of patients with concomitant biologics with “no” and “single or multiple, can be passed” for stenosis. While it did not reach statistical significance.

In conclusion, the present study demonstrated the role of BAE in the endoscopic assessment of disease activity and the predictive value for the risk of surgery in patients with small-bowel CD. A small-bowel SES-CD of ≥ 7 and stenosis based on BAE were associated with an increased risk of surgery. Based on the disease activity assessed by BAE, in small-bowel CD patients who have indicators of an increased risk of surgery, close monitoring and timely intervention need to be considered.

## Data Availability

Data underlying this article are available from the corresponding author upon reasonable request.
